# Revealing cancer subtypes with higher-order correlations applied to imaging and omics data

**DOI:** 10.1186/s12920-017-0256-3

**Published:** 2017-03-31

**Authors:** Kiley Graim, Tiffany Ting Liu, Achal S. Achrol, Evan O. Paull, Yulia Newton, Steven D. Chang, Griffith R. Harsh, Sergio P. Cordero, Daniel L. Rubin, Joshua M. Stuart

**Affiliations:** 1 Biomedical Engineering, University of California, Santa Cruz, USA; 2 UC Santa Cruz Genomics Institute, University of California, Santa Cruz, USA; 3 Stanford Center for Biomedical Informatics Research and Biomedical Informatics Training Program, Stanford University School of Medicine, Stanford, USA; 4 Stanford Institute for Neuro-Innovation and Translational Neurosciences, Stanford University School of Medicine, Stanford, USA; 5 Institute for Stem Cell Biology and Regenerative Medicine, Stanford University School of Medicine, Stanford, USA; 6 Departments of Neurosurgery, Stanford University School of Medicine, Stanford, USA

**Keywords:** Molecular subtyping, Community detection, MRI, Magnetic resonance imaging, Clustering, Mutation, Cancer

## Abstract

**Background:**

Patient stratification to identify subtypes with different disease manifestations, severity, and expected survival time is a critical task in cancer diagnosis and treatment. While stratification approaches using various biomarkers (including high-throughput gene expression measurements) for patient-to-patient comparisons have been successful in elucidating previously unseen subtypes, there remains an untapped potential of incorporating various genotypic and phenotypic data to discover novel or improved groupings.

**Methods:**

Here, we present HOCUS, a unified analytical framework for patient stratification that uses a community detection technique to extract subtypes out of sparse patient measurements. HOCUS constructs a patient-to-patient network from similarities in the data and iteratively groups and reconstructs the network into higher order clusters. We investigate the merits of using higher-order correlations to cluster samples of cancer patients in terms of their associations with survival outcomes.

**Results:**

In an initial test of the method, the approach identifies cancer subtypes in mutation data of glioblastoma, ovarian, breast, prostate, and bladder cancers. In several cases, HOCUS provides an improvement over using the molecular features directly to compare samples. Application of HOCUS to glioblastoma images reveals a size and location classification of tumors that improves over human expert-based stratification.

**Conclusions:**

Subtypes based on higher order features can reveal comparable or distinct groupings. The distinct solutions can provide biologically- and treatment-relevant solutions that are just as significant as solutions based on the original data.

**Electronic supplementary material:**

The online version of this article (doi:10.1186/s12920-017-0256-3) contains supplementary material, which is available to authorized users.

## Background

Expression-based subtypes have shown to be of tremendous use in predicting patient outcomes (e.g. PAM50 and MammaPrint subtypes for breast cancer prognosis) [1]. Most recently, transcriptome-wide RNA sequencing data or other high-throughput measurements have been used to segregate patient samples, which in turn has led to suggestions for changes in treatment of many cancers [2].

Both the sparsity of mutations and mutual exclusivity common in mutation profiles (within the same molecular pathways) [3] present challenges in the use of somatic variants for subtyping because similarities computed from the original mutation events lack specificity and robustness, due to the small number of overlapping events between any two samples. Subtyping patients based on magnetic resonance (MR) imaging data has shown promise (see [4] for a recent example). In such studies, the sparseness of anatomical/spatial MR image data, resulting from tumors occupying only a fraction of the imaged brain, can create issues for sample clustering.

**Fig. 1:**
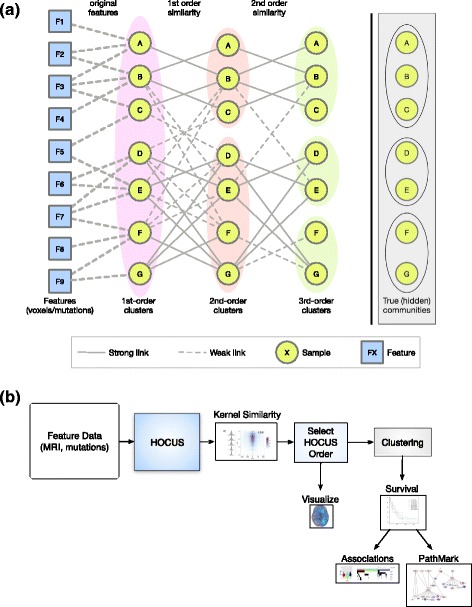
**a** Social network approach to clustering patient samples. First we transform/encode the mutation/voxel data, then compute all patient–patient similarities. At each order of similarities, clustering is based on similarities in that order, resulting in different clustering solutions. Shown here from left to right: features, 1st-order, 2nd-order, 3rd-order, ‘true’ communities. Note that links between the same sample but different orders are not shown (e.g. A always has a strong self link), but are used in the similarity calculations. **b** Flow diagram of HOCUS analysis: Feature data (such as imaging voxels or mutations are supplied to HOCUS to generate higher-order features from which sample-sample similarities are calculated. The HOCUS order is selected by comparing sample-sample similarity kernels with an external criterion. Clustering is then done followed by survival and downstream analyses

In this work, we evaluate the use of “higher–order” similarity measures between the samples to identify biologically relevant subtypes. Intuitively, higher–orders compare two samples based on how similar their sample “neighborhoods” are to one another rather than using the original data. Comparing neighbors can provide a broader perspective on what might otherwise appear to be a coincident similarity: a low level of overlap between sparse feature sets may be relatively specific for a particular subset of samples. To illustrate, consider the toy network shown in Fig. 1. A first-order network links any two samples with some minimum first-order similarity. A second-order network then links samples with overlapping neighborhoods in the first-order network. Repetition of this procedure generates higher-order networks from a lower-order version, that could reveal community structure. By the third order, the three communities emerge despite the fact that the original features are only weakly associated with the ground truth clusters.

We use such a similarity transformation, here referred to as Higher-Order Correlations to Uncover Subtypes (HOCUS), and show that HOCUS enhances the detection of biologically-relevant clusters of patients for several Cancer Genome Atlas (TCGA) cohorts. HOCUS is applied to both categorical and ordinal data modalities, using mutation and copy number data. In addition, we apply HOCUS to Gadolinium- based contrast-enhanced T1-weighted preoperative axial MRI data and establish links between MR image features and patient overall survival.

We looked for inspiration from fields with similar data, such as social network analysis. Detecting community structure is an important problem in the study of many different types of networks including social (e.g. connected friends), online (linked web pages), and molecular (regulatory gene signaling). In these applications, communities represent sets of densely connected nodes within a larger set of nodes in a network. Cliques of friends with shared interests or a gene module representing the shared function of genes in a biological pathway are examples of such communities.

Community detection techniques have so far been under-utilized for the purpose of subtyping patients based on shared genomic- and image-based events. Yet the application is straightforward – the data can be converted readily into a network of patient samples using sample-sample similarities. Mutations and MR images are examples of sparse data, since few mutation events are shared between patients and since the relative ratio of tumor to normal tissue in the brain means that most regions are tumor-free and so patient tumors are rarely in the same location as other patient tumors. We asked whether higher-order comparisons could boost performance when clustering these data. We find several cases in which the HOCUS community detection approach identifies biologically meaningful patient subtypes that can be distinct from those identified using the direct features.

## Methods

### Overview of HOCUS clustering

HOCUS uses a technique from network analysis in which samples are compared based on their neighborhood similarity [5–7] and can be pictured as the construction of progressively higher-order networks (Fig. 1). Features are somatic mutations, copy number events, or 3D MR brain images of patients. Sample-sample similarities were calculated using an appropriately chosen similarity metric (Additional file 1: Figure S1, Section 0.0.2) that can be viewed as a sample-by-sample network. Higher-order similarities were derived from lower-order similarities by treating the lower-order similarities computed at step *k* − 1 as the features used to compute new similarities at step *k*, similar to Yu et al. [8] and Yu et al. [9], and to multiplying a network adjacency matrix to itself one or more times in order to reveal connected components linked by reachable paths. The samples can be clustered using either the original features (i.e. use first-order similarities) or features derived from higher-order similarities, identifying groups of patients having a higher proportion of transitive relations.

### MR images

MR Imaging data were downloaded from the Cancer Imaging Archive (www.cancerimagingarchive.net), and were processed using Slicer [10]. Tumor location was extracted from T1 anatomic MR images as previously described [4]. Patient tumor was identified in the MR images by having two experts delineate tumors’ regions of interest, were registered to a common coordinate space of a brain atlas along with anatomical T1 brain images to obtain registered tumor ROIs, and then feeding through the image-processing pipeline developed in an earlier paper by some of the current authors [4]. This results in a per-patient 3-dimensional binary matrix of tumor-containing and tumor-free voxels (3-dimensional pixel) in the brain. Each 1 mm MR image slice was rotated and aligned to a brain atlas (Montreal Neurological Institute 152 [11]), to make voxels comparable between patients.

### Mutations

Mutation and copy number data were downloaded from Firehose (firebrowse.org); BRCA, GBM, and OV data were downloaded March 18,2015; BLCA data were downloaded Nov 9, 2015; and PRAD data were downloaded March 4, 2015. Only non-silent mutations were retained for mutation-based analyses. In other words, mutations in locations that were predicted as deletions, insertion, splice-site, missense, or nonsense mutations, were kept. These data were recorded into a binary-valued patients-by-genes matrix. Only genes with at least one non-silent mutation within the cohort and patients with at least one non-silent mutation were represented in the matrix.

### Copy number

GISTIC2 [12] copy number variation data was downloaded from Firehose (firebrowse.org). Sample-sample similarities were derived from Hamming distances computed on the GISTIC integer-valued copy number estimates ([−2,−1, 0, 1, 2]). Thus any identical copy number values between tumor genomes are considered a match in the similarity calculation.

### Visualization of conditional densities

To visualize the association between feature- and survival-based measures, we plotted the proportion of sample pairs with similarities in both metric spaces. If the distribution of survival similarities for sample pairs changes as a function of the feature-derived similarities, it suggests that the feature-based metric carries outcome-relevant information. For example, if we restrict the pairs to those with high kernel similarity in mutation space and we observe that there are more pairs with similar survival compared to the background (or to pairs with low mutation-based similarity) it would indicate mutation-based similarity carried information about survival outcome. To view such a dependency, we group sample pairs into bins of approximately equal feature-based similarity. Then, for each bin, we plot the distribution of outcome similarities, shown along the left-hand side of each conditional density plot. A distribution that changes significantly across the bins reflects an association between the feature- and outcome-based similarities. In the case of patient survival, we are interested in whether larger similarities computed from the feature data reveal a larger proportion of patient pairs with similar survival times.

### Community detection using higher-order sample similarities

Our analysis is related to the common inference-by-transitivity technique used in social networks, summarized by the statement ‘a friend of my friend is also my friend.’ This technique finds cliques of similar patients in a network by connecting patients that are similar in the original network and then clustering based on those similarities. Given samples *j* and *k*, and feature vectors *X*, we calculate the similarity matrix *S* ^(1)^ (using Hamming similarity (*S*_*H*_^(1)^) when the features are binary such as for mutations and imaging voxels).

Where *n* is the number of features (e.g. voxels), *I*(*a*, *b*) is the indicator function that returns *1* if its first argument equals its second and returns *0* otherwise. Using this similarity metric, we compute the 2nd-order similarities from the 1st-order matrix. Let *m* be the number of samples in the cohort. The second-order metric is calculated as:

1 $$ {S}^{(2)}:{S}^{(2)}\left( j, k\right)=\frac{\frac{1}{n}{\displaystyle {\sum}_{l=1}^m}\;{S}^{(1)}\left( j, l\right)\ {S}^{(1)}\left( l, k\right)}{\sqrt{{\displaystyle {\sum}_{l=1}^m}\;{S}^{(1)}\left( j, l\right)}\cdot \sqrt{{\displaystyle {\sum}_{l=1}^m}\;{S}^{(1)}\left( l, k\right)}}, $$

which is just the definition of the correlation between the *j* th row and *k* th column in *S* ^(1)^ matrix. For higher-order clustering, the precomputed similarity matrix is raised to the *d* power, where *d* is the order of clustering.

Consensus clustering subsamples both the features and samples in the data. Each subset of the data is then clustered using a user–specified clustering algorithm (in this paper, *k*-means clustering). The process is repeated for a user–specified number of iterations (we used 1,000). A consensus matrix is constructed based on the proportion of iterations in which 2 samples are clustered together. The final cluster assignments are based on the consensus matrix. ConsensusClusterPlus clusters matrix *S* using a user–specified range of number of clusters (in this case up to 10 clusters) and the ‘best’ solution is chosen based on average Silhouette score and on the relative change in the cumulative distribution function (CDF) scores calculated by ConsensusClusterPlus. CDF scores are used to find the number of clusters at which there is maximum stability between the subsampled clustering solutions, indicating that the clusters are representative of true clusters in the data.

Because the ConsensusClusterPlus R package [13] computes an internal metric prior to clustering and only takes as input a feature matrix, we would raise the matrix to the (*d* − 1) th power and supply this as input to ConsensusClusterPlus, as the feature matrix. Using centered Pearson Correlation as the metric is then equivalent to squaring the feature matrix. In this way, we tested all even powers of *d* when using ConsensusClusterPlus. For example, our “third-order” solution effectively uses a fourth-order metric since *S* ^(2)^ is squared and our “fourth-order” solution is actually a sixth-order metric since *S* ^(3)^ is effectively squared inside the ConsensusClusterPlus package. In this paper, HOCUS orders refer to the iteration and not the power of the matrix.

## Results

We applied HOCUS to the problem of detecting cancer subtypes using two very different data modalities – somatic mutations and 3D tumor imaging data. Clustering patient samples by their shared genomic events or related imaging features may reveal common disease etiology important for outcome assessment. Yet mutation and imaging data are sparse – sample pairs have few overlapping events. It is therefore problematic to use these data as features directly for clustering since similarities calculated from sparse spaces suffer in sensitivity and specificity [14]. Similarities based on the local neighborhood in a network can be more sensitive because this approach can capture samples having an indirect coincidence through other samples. We show that the use of HOCUS for either mutations or imaging data adds specificity as it produces inferred subtypes that are biologically– and clinically–relevant that were undetected by the approaches using lower-order metrics.

### Community detection reveals cancer subtypes using somatic mutation data

The particular ways in which a tumor genome is altered creates a signature that reflects the type of cell and mutagens involved. Driving events involving specific genes are associated with certain cancer types. For instance, BCR-ABL fusions are characteristic of chronic myeloid leukemia. The question is whether the pattern of mutations within these cells-of-origin can further subdivide the patient samples into meaningful categories that may be associated with patient outcomes.

We applied HOCUS to mutation data for 3 TCGA cancers: high-grade serous ovarian cancer (OV), glioblastoma multiforme (GBM), and bladder urothelial carcinoma (BLCA). We computed Hamming similarity over the mutation data for n genes as:

2 $$ {S}^{(1)}:{s}^{(1)}\left( j, k\right)=\frac{1}{n}{\displaystyle {\sum}_{i=1}^n\; I\left({x}_{i, j},{x}_{i, k}\right),} $$

where *j* and *k* denote two different samples, and *x*_*i*,*j*_ is the value of feature *i* for sample *j*. The Hamming distance counts the fraction of matching mutated genes for all sample pairs, resulting in an adjacency matrix of *m* × *m* samples. We retained for clustering all metrics that provided a non-redundant set of relations between samples not captured by lower-order metrics. For higher-order clustering, we raised the precomputed similarity matrix *S* to the *d* − 1 power, where *d* is the order of clustering. We then supplied this similarity matrix as the feature matrix for input to consensus clustering (see Methods). Figure 1 shows a conceptual example of this principle– as the order of clustering increases, cliques in the network emerge and form clusters.

To do this, we identified all *k* ^th^-order metrics and lower such that the (*k* + 1)^st^ metric produced highly similar relative similarities to the kth metric as measured by a kernel alignment test [15], (see Additional file 2: Figure S2). We sought to determine if higher-order feature-based similarity measures (i.e. those based on mutations, images etc) had an enrichment for connecting patients with similar survival outcomes compared to using first-order feature-based measures.

**Fig. 2:**
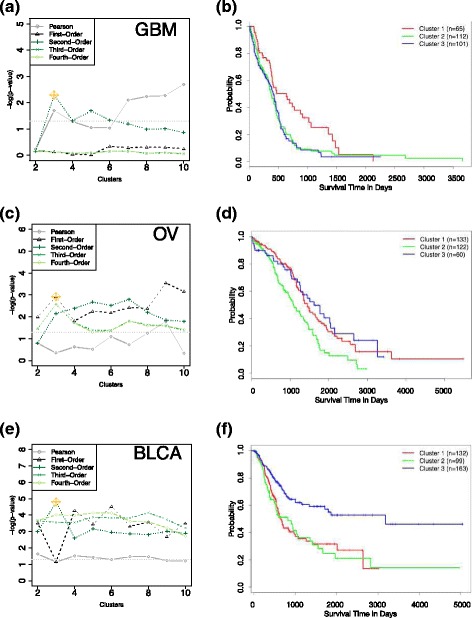
HOCUS in first- through fourth-orders, and Pearson clustering of **a** GBM **c** OV and **e** BLCA survival *p*-values (log-rank test) vs number of clusters. **b** GBM, **d** OV, and **f** BLCA Kaplan-Meier plots for selected HOCUS clustering solutions (starred in yellow on the survival *p*-value plots (**a**, **c**, **e**). Clusters with fewer than five samples are excluded from the KM analyses

For each tumor type, we clustered the patient samples based on either Pearson correlation, the first-order Hamming similarities, or non-redundant higher-order similarities. We used K-means consensus clustering [13], for K = 2 to 10, and calculated the degree to which the solutions separated patients with different outcomes as a measure of biological relevance. A Kaplan-Meier test was performed on each clustering solution and the significance (-log *P*-value, log-rank test) was recorded (Fig. 2).

We applied HOCUS to the TCGA GBM dataset containing 283 patients for which 14,910 mutations were found across 7,874 distinct genes, and found 3 distinct clusters. Survival differentiation has proven difficult to achieve in previous analyses of GBM datasets [16, 17], however the HOCUS results show some difference in survival between clusters. Note that Pearson correlation achieves greater difference in survival but must consider at least ten clusters, which is likely an over-partitioning of the samples for this data set. Patients in the best surviving cluster, cluster 1, had low EGFR and TTN mutation occurrence compared to that of patients in other clusters; TTN mutations are predominantly in cluster 3 and EGFR mutations distributed between clusters 2 and 3. All 14 of the IDH1 mutated tumors were in cluster 1, as were most (11 of 16) of the ATRX mutants. The cluster corresponds well with mRNA cluster 3 (LGr3) from the recent TCGA paper [18]. Thus, the HOCUS clustering using mutations seems to have been able to tease out a low grade diffuse subtype, defined by IDH1 mutation status seen in younger individuals, characterized by the absence of a 1p/19q codeletion and a lack of TERT expression and an overall better prognosis. Furthermore, all 65 samples in cluster 1 have a TP53 mutation (Additional file 3: Figure S3), whereas there are none in cluster 2 and only 14 (of 105 samples) in cluster 3.

We next applied HOCUS to the TCGA OV dataset containing 316 patients for which 14,810 mutations in 8,258 genes were reported by the TCGA analysis working group. For the OV dataset, the first-order solution found the greatest separation in survival between clustered groups. Higher-order metrics gave different solutions but were comparable with the first-order solution in separating out patient groups with differences in outcome. One of the main divisions of the samples shows a significant difference in overall mutation rate (Additional file 4: Figure S4). In addition to TP53 mutations, several genes that are characteristic of passenger mutations were also predominant in the highly mutated cluster including TTN, MUC16, and RYR2. Other mutations were significantly associated with these clusters, highlighted in Additional file 4: Figure S4. HOCUS OV clusters correlate with platinum resistance, which is a survival marker.

These findings were surprising given that the TCGA OV dataset has posed a significant challenge for analysts to identify meaningful genome-based distinctions between the patients [17, 19]. One of the most successful attempts to date was reported by Hofree et al. [20] in which patient samples were clustered based on a network diffusion transformation of the mutation data. To compare the two approaches we ran HOCUS using the TCGA OV data as filtered by Hofree et al., whereas we do not filter the mutation datasets. Our results indicated that comparable survival differences to the NBS approach could be obtained by using a different metric (e.g. Hamming distance used here) and higher-order HOCUS, eliminating the need to introduce prior knowledge (Additional file 5: Fig. S5). A similar result was obtained when applying HOCUS to a TCGA breast cohort (see Additional file 6: Section 0.0.4) in which both the first-order and second-order results revealed similar survival separation while producing different clustering solutions. Thus, since the first and second-order solutions for both OV and BRCA (Additional file 7: Figure S6, Additional file 8: Figure S7, Section 0.0.4) gave different clustering solutions but comparable outcome separation, it is possible that a solution combining first- and second-order solutions could produce a better outcome predictor for the patients. Furthermore, since HOCUS performed better on the OV dataset when hypermutated samples and hypomutated genes are excluded from analysis, it would be beneficial to experiment with more extensive data preprocessing.

**Fig. 3:**
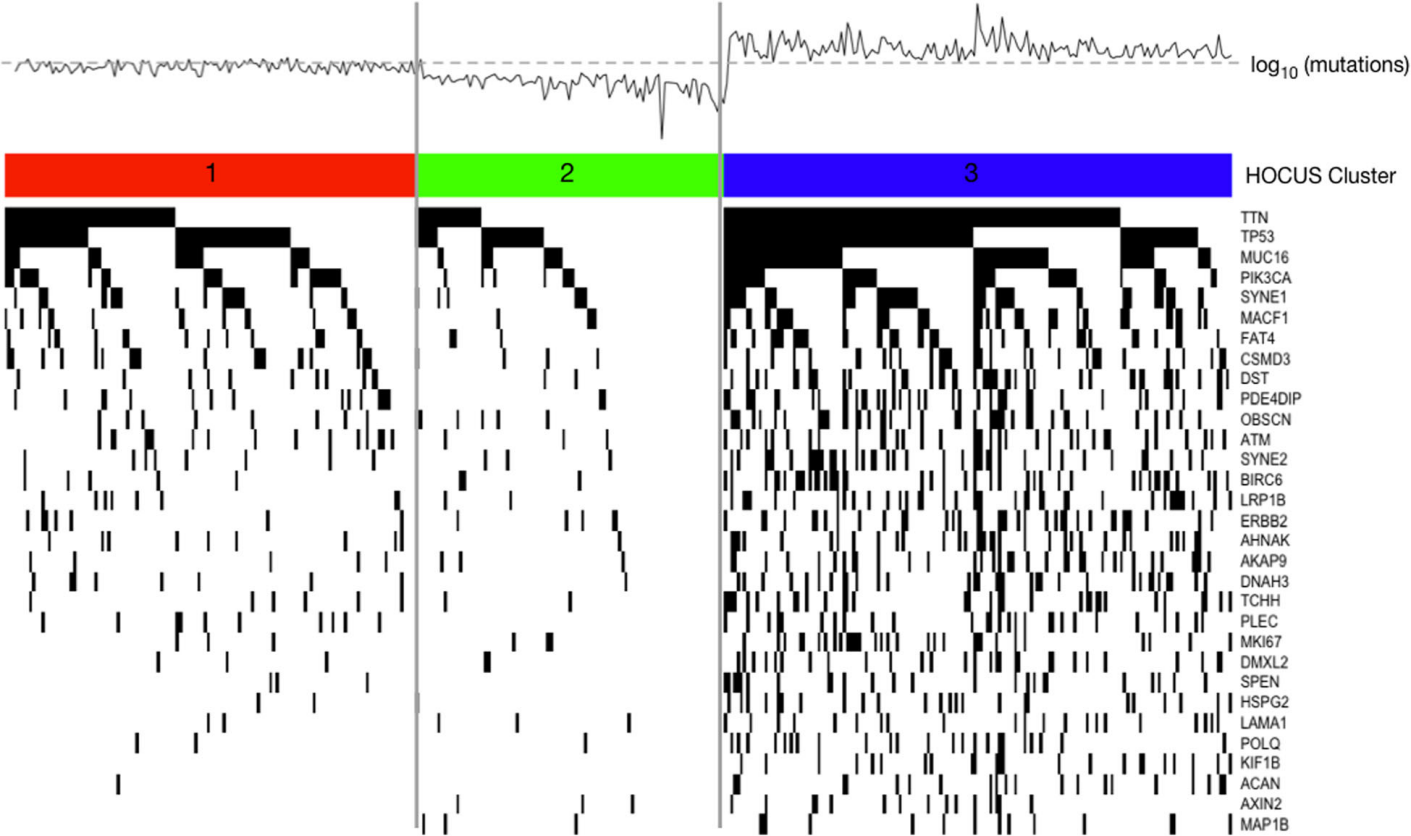
Oncoprint showing a subset of mutations in BLCA. Line plots above the oncoprint show the total number of mutations per sample. The grey dotted lines indicate median mutational frequency across the cohort. This BLCA oncoprint includes genes with the smallest *p*-values in a *χ* ^2^ test of independence when compared to mutation rates outside the cluster. We compared each cluster to all others combined

We next applied HOCUS to the TCGA BLCA cohort of 394 patients for which 84,048 mutations were called based on exome sequencing, covering 15,553 distinct genes. HOCUS uncovered distinct clusters in BLCA. Compared to all HOCUS orders, 2nd–order has the largest separation in survival of the clustered patients. We note that, like the case for OV, the clusters are associated with the number of mutations per sample. Indeed, clustering by mutation rate alone yields comparable separation in patient outcomes (log-rank test, *P* <4^−10^ 5; Additional file 6: Figure S8) as the HOCUS solution. Since mutation data was the only data used, we searched for genes with mutations that discriminate the patient clusters to understand the different underlying etiologies. Figure 3 shows the top 15 genes associated with each cluster using a *χ* ^2^ test of independence (due to overlap in the ‘top’ genes for each cluster, 31 genes are shown). Many of these genes were associated with several cancer types, for example LRP1B has been associated with thyroid, ovarian, renal, and brain cancers [21–23]. Other known oncogenes such as PIK3CA (*χ* ^2^ test, *p*-value 3.6 × 10^−4^) and TP53 (*χ* ^2^ test, p-value 3.9 × 10^−8^) are also significantly associated with the clusters. Interestingly, the highly mutated BLCA cluster has the best survival prognosis. On average, cluster 3 patients had a 1.7 to 2.2 times higher survival probability at the 5-year mark (0.56 compared to 0.33 for cluster 1 and 0.25 for cluster 2). The 95% confidence interval of the survival probability at 5-years for cluster 3 patients was (0.45, 0.66) compared to (0.12, 0.38) for cluster 2 patients and (0.22, 0.43) for cluster 1 patients.

In both cases, a higher rate of TP53 mutations was found (80% compared to the background rate of 50%), and a slightly higher rate of smokers was in the category. However, when we compared our clusters to the TCGA BLCA clusters, which were generated using mutation and copy number data with an integrated NMF approach, we found only a weak correspondence (Additional file 9: Figure S8(b), *χ* ^2^ test, *p*-value 0.128). Thus HOCUS finds a solution that is distinct from the TCGA–derived subtypes and one that has a comparable association with survival outcomes.

We applied HOCUS to the TCGA Pancancer-12 mutations data. Clusters were derived for 3,394 samples using 313 mutated genes (listed by recent TCGA studies as high–confidence driver mutations [24, 25]) from which similarities were calculated. We visualized the resulting clusters with the Tumor Map tool (https://tumormap.ucsc.edu/?p=Pancan12.SampleMap&li=5). Tumor Map projects the HOCUS 2nd-order sample–sample similarities onto a 2-dimensional map based on the Google maps framework. Tumor Maps built from mutation data using Pearson correlations failed to separate samples into distinct clusters (data not shown), whereas the HOCUS clusters correlated with many other genomic features and were able to find subtypes among the samples (Additional file 10: Figure S9). The subtypes are characterized by clusters of samples from many tissues of origin. Not surprisingly, the main difference in most of the clusters is whether they contain samples with either a TP53 or PIK3CA mutation. However, of note is that HOCUS found a pancancer cluster (dashed boxed in Additional file 10: Figure S9) with samples containing both TP53 and PIK3CA mutations. These tumors either represent cases in which these two frequently mutated genes are altered or consist of tumors with higher subclonal heterogeneity that contain distinct subclones of either TP53 or PIK3CA mutated cells. In either case, these tumors would be of particular interest as they harbor two major drivers of oncogenesis. An attribute enrichment analysis revealed that samples clustered into the TP53-PIK3CA group are composed of tumors in later stages on average than those outside the cluster, consistent with a longer and more complex evolutionary history for these samples.

In summary, when applied to tissue-specific cohorts, the mutation-based HOCUS subtyping reveals distinct sample groupings that appear to be as biologically significant, based on survival comparisons, as the original TCGA expression-derived subtypes, which have been shown to be highly correlated with histopathology calls. On the other hand, when applied to a diverse collection of tumor types that span multiple tissues, the HOCUS-based mutation clusters identify major divisions that are not primarily tissue based. Taken together, this suggests HOCUS could be used in a classification system of the tumors in which mutation data is incorporated, along with other data like copy number and gene expression. Rather than considering mutated genes independently in this process, as was recently done to build pancancer decision trees for this data [26], HOCUS clusters could be used instead, providing potential mutated gene combinations for division points like the TP53- PIK3CA subtype described here.

### Community detection of subtypes using copy number data

To test the applicability of HOCUS to other data, we also applied the technique to the clustering of patients based on Hamming similarity of GISTIC2 discretized copy number data (see Methods). We applied HOCUS to TCGA prostate adenocarcinoma (PRAD) copy number data because prostate cancers are known to harbor significant copy number events over the evolution of the tumor including AR amplifications, TMPRSS2-ERG fusions, and even whole genome level events such as chromoplexy. We converted the continuous copy number data to ordinal by using the output of the Broad’s GISTIC2 pipeline [27] that provides gene-level associated copy number estimates. GISTIC2 scores indicate copy number aberrations, where 1 indicates low-level and 2 indicates high level amplifications, negative scores indicate the same but deletions rather than amplifications, and a score of 0 indicates no copy number alterations. For the TCGA PRAD cohort, survival rates are sufficiently high making patient survival time an inappropriate measure of disease subtype. HOCUS cluster 2 patients have higher Gleason scores, more lymph node invasion, and higher stage (Additional file 11: Table S1, Additional file 12: Figure S10), all of which are associated with disease aggression.

### Community detection from magnetic resonance imaging data

We next applied the HOCUS method to the task of grouping patients with GBM based on the imaging of their tumors. Previous imaging studies using MRI have extracted location and anatomical features to characterize tumors. Recent imaging studies have utilized MR images to define patient subtypes for personalized treatment [4, 28, 29]. MR images are 3-dimensional and contain millions of pixels and human brains have variable size and shape, making it difficult to compare patients. By mapping the MR images to the MNI brain atlas (Montreal Neurological Institute 152 [11]), we were able to compare patient images, and using HOCUS we were able to find clinically relevant imaging subtypes.

We applied HOCUS clustering using the GBM voxel (3-dimensional pixel) data from the TCGA collection of 184 patients with first- and higher-order metrics to find community structures. MRI data are part of the TCGA GBM cohort, downloaded from the Cancer Imaging Archive and processed by Stanford University as described in Liu et al. [4]. To reduce noise and the size of the MR images, we first preprocessed the data by filtering to a set of informative voxels containing tumor in some, but not all, of the patients (Additional file 13: Figure S11). We removed subsequent analysis all non-informative voxels with tumor in fewer than 15 individuals (see Additional file 6: Sec. 0.0.1). We computed sample-to-sample similarities using the remaining voxels and performed higher order calculations and clustering as described above for the mutation data (e.g. Hamming distance and ConsensusClusterPlus were used). Cluster solutions revealed that the metrics converged by the fourth-order (Additional file 14: Table S2).

We sought to determine which metric based on the imaging data best matched up with the observed differences in patient outcomes. We defined an outcome-based similarity metric by computing all pairwise absolute differences between the survival time of every pair of patients, *d*_*ij*_ = |*T*(*i*) − *T*(*j*)|, where *T*(*i*) is the survival time in days of patient *i*. These distances were converted to co-survival similarities via the linear transform ${s}_{ij}=\frac{1}{m}\left(1-{d}_{ij}\right)$, where *m* = *max*{*d*_*ij*_} is the maximum absolute differences between any two patients. We then quantified the correlation between imaging-based similarities and co-survival similarities using a normalized version of the kernel alignment method of Cristianini et al. [15] that calculates a centered correlation between two full sample-by-sample similarity matrices. We repeated the kernel alignment comparison to co-survival for first-order and higher order HOCUS metrics (Additional file 14: Table S2).

**Fig. 4:**
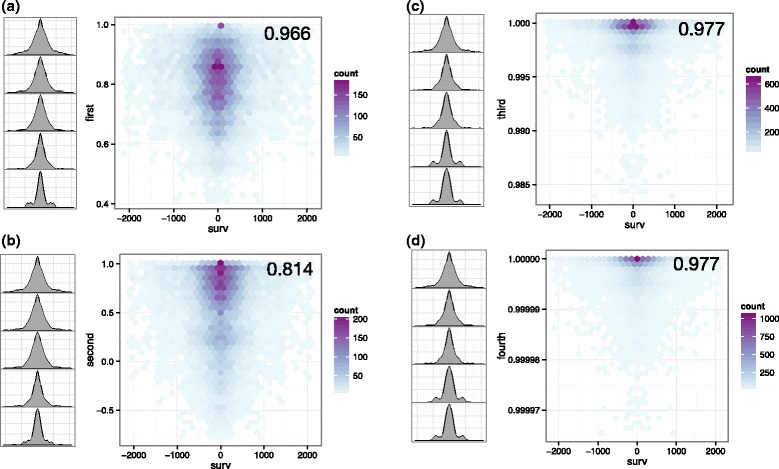
Visualization of sample pair frequencies of image-based metrics compared to survival outcome metric; results on the **a** first-order, **b** second-order, **c** third-order, and **d** fourth-order HOCUS. For this visualization only, data was restricted to patients with a death event, then sample–based pairwise correlations were calculated using 1st-, 2nd-, 3rd-, 4th-order HOCUS metrics as well as difference in the length of survival, in days, between each pair of patients. In each plot, the conditional density is shown in which the distribution of all sample pairs are depicted as density maps. On the left-hand side of each plot, a series of plots are shown in which the feature-based measure is divided into five bands of equal size, and differences in survival time (the outcome metric) are plotted in histograms for those samples restricted to each band. The kernel similarity between the HOCUS metric and the co-survival metric is shown in the top right corner of each plot

To visualize the results of the kernel alignment comparisons, we plotted the probability densities (Fig. 4). The third- and fourth-order had the highest kernel similarity scores to co-survival as can be verified visually. We chose the third–order solution because fourth–order produced similar groupings to the third, and thus the added complexity of using a higher order was not justified in this case. Interestingly, second-order had a lower kernel similarity to co-survival than first-order, illustrating the benefit of searching several higher order metrics beyond 2nd–order. We note that second-order HOCUS stratifies MR images into tumor groups by anatomic location (Additional file 15: Figure S12(a)). On the other hand, third-order clusters were driven by a combination of location and volume. In addition, third-order produced a larger separation of survival in groups than location or volume alone.

**Fig. 5:**
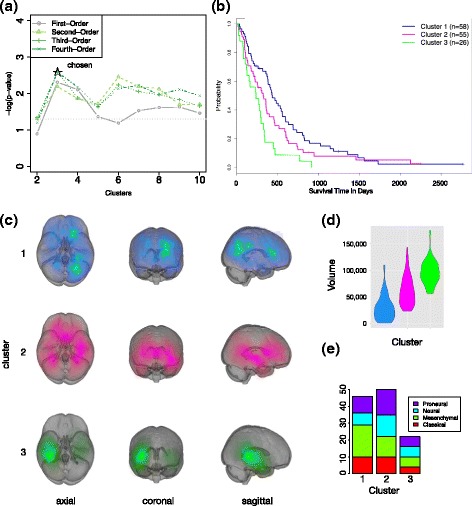
HOCUS of GBM MR Images. **a** *P*-values of survival separation (log-rank test) for each of the orders of clustering across a range of *k* clusters. **b** Kaplan-Meier plot of the third-order HOCUS clusters. **c** Images of tumors within each cluster projected onto the MNI brain atlas. Showing sagittal, coronal, axial views. Brightness of color indicates the number of patients with tumor at a given location. Generated using Slicer [10]. **d** Violin plot showing tumor volumes within each third-order cluster. **e** Molecular (gene expression based) subtypes within the clusters

Each clustering solution that is based on a different metric order identifies different characteristics in the MR images that are associated with survival prognosis. While first-order clusters, based on Hamming distance, align with tumor volume, and second-order with anatomic location (Additional file 15: Figure S12), third-order clustering captures aspects of both tumor volume and location (Fig. 5(c-d)). Each solution has statistically significant separation in survival (Fig. 5(a)), with third-order having the greatest separation in survival of image cluster groups. Patients with tumors in the frontal lobe and which are smaller in volume had significantly better survival than larger tumors in the lower rear portions of the brain.

**Fig. 6:**
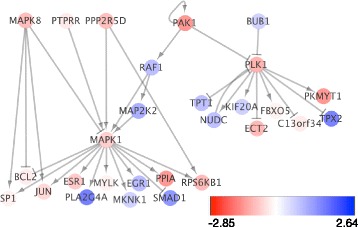
PathMark analysis of the poor surviving third-order cluster vs others. Node size and color indicates differential expression levels

Interestingly, the third-order solution pulled together patients that made up two separate poor surviving clusters in the second-order solution. To better understand the third-order subtypes revealed by the imaging data, we inspected the genetic pathways that distinguish the poorer surviving subtype from the others using RNASeq gene expression data available for 184 patients. We computed a differential expression score for each gene to indicate whether a gene’s expression level was higher or lower on average in the poorer surviving cluster (cluster 3) relative to the others using the Statistical Analysis of Microarrays technique [30]. We then connected any gene with an absolute differential expression higher than one standard deviation above the average of all genes. Finally, we retained pathway interactions connecting only those genes that were both in this set and plotted them with the Cytoscape viewer [31]. Several pathways involved in major growth and proliferation signaling were implicated from these networks as might be expected (Fig. 6). ERK (MAPK1) was found to be significantly overexpressed in cluster 3 tumors along with JUN-kinase (MAPK8). In addition, AKT1 and PLK1 were also found to be higher in cluster 3, both known to drive cell cycle progression.

## Discussion

To explore how patient-to-patient similarity transformations influence subtyping, we introduced a method called Higher-Order Correlations to Uncover Subtypes (HOCUS) that iteratively calculates higher order metrics using each similarity space to define patient clusters. HOCUS uses network connectivity to define groups or ‘communities’ of patients, related by both direct and indirect connections, reinforced by transitive relations in a local subnetwork. The higher-order metrics incorporate information from local neighborhoods to assess if two patient samples are related. In several cases we find that HOCUS provides an improvement over methods that use the molecular features directly to compare samples (Fig. 2). We find that higher order metrics yield better clusters for BLCA and GBM patients based on mutations, as well as GBM patients based on their tumor images.

In the case of BLCA cancer, the second-order metrics revealed groupings of the patients where tumors with higher mutation rates are separated from the other tumors and these patients have an overall better survival outcome. Most notably, the solutions for BLCA and OV separate tumors with higher mutation rates from the others and those patients with higher mutated tumors have a better survival outlook relative to the other patients. This result may reflect that highly mutated tumors are more sensitive to DNA damaging agents (e.g. cisplatin treatment for OV patients). Alternatively, a higher mutation rate could increase the number of neo-antigens present on tumor cell surfaces, helping a patient’s innate immune system to identify and eliminate tumor cells that lack immunosuppressive protection such as through the expression of PD-L1 and/or CTLA4. Consistent with this idea, recent clinical trials have found that combining DNA damaging agents with immunotherapies can have synergistic effects [32]. Alternatively, tumors with higher mutation rates could reflect a different subtype with an intrinsically distinct progression pattern. In support of this, we do find a somewhat higher proportion of papillary BLCA tumors in the higher mutated cluster (44% of papillary BLCA tumors are in cluster 3), but this association is not significant based on a *χ* ^2^ test.

Medical images are an underused resource that have vital information [33–38]. A key piece of data conveyed by imaging is the location of a tumor in the brain, which can influence when the tumor is detected due to the tumor affecting certain location-associated brain functions. For example, some tumors may be detected early because they reside in regions that induce extreme nausea in patients. Furthermore, some tumor locations may be more resectable than others, such as the frontal lobe and surface [39]. Thus, imaging data carries important aspects for subtyping patients according to disease outcome and treatment selection. In the TCGA data, molecular subtype is independent of both tumor location and volume (Additional file 15: Figure S12(c), Additional file 16: Figure S13).

HOCUS clustering using GBM imaging data automatically clustered the patients into groups by anatomic tumor location. Our work corroborates that of others in finding regions associated with poor survival in GBM patients [4, 40, 41]. We highlight the benefits of using both genomic and image data in predicting cancer progression, as imaging data on its own has little predictive power on its own (Additional file 17: Figure S14), and recommend combining them in future analysis. We also show how location influences genomics in GBM as an independent covariate of molecular subtypes. Both image and genomic data are key to understanding GBM. Of note, IGF1 was found to be the most differentially expressed gene in cluster 3 (Additional file 18: Table S3). Higher levels of IGF, or its receptor, could point to an alternate metabolic requirement for these tumors. It would be interesting to follow up on this observation by testing if the receptor is present on tumor cell surfaces to support the possible role of this growth pathway. If tumor growth is dependent on this pathway than blocking IGF receptor activity may show benefit in these patients.

## Conclusions

As demonstrated here, community detection approaches may have merits for subtyping patients when using sparse data (few events in any single patient sample). We introduced a visualization method to augment the quantitative kernel alignment for identifying when a similarity measure is associated with an outcome measure of interest. The visualization inspects the distribution of the patient outcome similarities as a function of the feature-based similarities. In several cases tested, the procedure revealed that a higher-order metric was more associated with survival than non community-informed metrics. This supports the notion of using community detection techniques for the analysis of genomics and imaging data, especially given sparse feature spaces. The question remains on how to identify the appropriate order and how many orders should be tested for a given study, with a given type of data set, especially in a situation where outcome based information is unavailable.

HOCUS is applicable to both binary (i.e. mutation and voxel) data and ordinal data (e.g. CNV). HOCUS is simple and flexible enough to be used wherever a suitable similarity metric between individuals can be generated, even for non-sparse data such as expression or methylation data. As we have shown, its application has the potential to reveal groupings missed when using standard metrics. HOCUS code is available at github.com/graim/HOCUS.

## Additional files

Additional file 1: **Figure S1.** Alternative similarity metrics used to compare patients. (a) *P*-values of survival differences (log-rank test) between clusters for each similarity metric over a range of clusters, (b) tumor volumes of second-order TFIDF clusters, (c) molecular subtypes within each second-order TFIDF cluster. (d) Tumors volumes of Jaccard clusters, (e) barplot of molecular subtypes by Jaccard cluster, (f) brain images of Jaccard clusters, and (g) a ribbon plot showing the changes in cluster membership when using different similarity metrics on the same data. (EPS 22877 kb)

Additional file 2: **Figure S2.** Visualization of association between mutation-based and outcome-based similarity measures for TCGA cohorts: a) OV, b) BLCA, and c) GBM. For this visualization only, data was restricted to patients with a death event, then pairwise correlations were calculated in each feature space (Pearson, 1st-, 2nd-, 3rd-order HOCUS) as well as difference in the length of survival, in days, between each pair of patients. A series of plots, one for each metric (Pearson correlation, hamming similarity, or higher-order) for three different tumor analyses. In each plot, the joint density is shown in which the distribution of all sample pairs are depicted as density maps. On the left-hand side of each plot, a series of plots are shown in which the feature-based measure is divided into five bands of equal size, and differences in survival time (the outcome metric) are plotted in histograms for those samples restricted to each band. The number in the top right corner of each plot is the kernel similarity between the HOCUS metric and the co-survival similarity. In every case tested, a higher-order metric could be found that had a positive association with the survival similarity metric, whereas Pearson correlation, based on the original features, had seemed to have a low and sometimes negative association. For example, the surprising negative association of the Pearson-based first-order measure is evident where most highly correlated sample pairs actually show an appreciable increase in samples with very different survival outcomes (seen as the introduction of extra ”modes” in the top histograms). For BLCA and GBM cohorts the higher-order clustering solutions revealed subtypes with better survival separation than first-order metrics. For OV, the higher-order metrics performed comparably with Pearson just outperforming. (EPS 159131 kb)

Additional file 3: **Figure S3.** (a) Violin plot showing mutational frequency per cluster and (b) Oncoprint showing a subset of mutations in GBM that are associated with cluster 1 via a *χ* ^2^ test and are mutated in at least 10 samples. Line plot above the oncoprint shows the total number of mutations per sample, and the grey line indicates median mutational load across the entire cohort. We show 5 frequently mutated genes that are associated with GBM mutations HOCUS result via a *χ* ^2^ test of independence, cluster 1. (EPS 348 kb)

Additional file 4: **Figure S4.** Oncoprint showing a subset of mutations in OV. Line plots above the oncoprint shows the total number of mutations per sample. The grey dotted lines indicate median mutational load across the cohort. A combination of the most frequently mutated genes in the OV cohort (colored black) and the genes significantly associated with any 1st-order HOCUS cluster through a *χ* ^2^ test of independence are shown. Colors in the oncoprint indicate which cluster the mutation is associated with. TTN, a known passenger mutation, is associated with clusters 2 and 3. (EPS 971 kb)

Additional file 5: **Figure S5.** Comparison to Network-Based Stratification [6] using the TCGA OV data used in their publication, and the same filtering. (JPG 528 kb)

Additional file 6: Supplemental text. (DOC 132 kb)

Additional file 7: **Figure S6.** Oncoprint showing the HOCUS BRCA clusters and associated mutations. (EPS 5745 kb)

Additional file 8: **Figure S7.** Visualization of the BRCA copy number clusters and their correlation with the mutation-based subtypes from HOCUS. Heatmap made using the UCSC Cancer Genomics Browser [7], showing TCGA CNV subtypes and CNV alterations in the HOCUS clusters. (JPG 531 kb)

Additional file 9: **Figure S8.** (a) KM plot where samples are grouped by overall mutational frequency. *P*-value 4.7e − 05 (compared to HOCUS *p*-value 1.59e − 05), and (b) Alluvial diagram showing the difference in HOCUS 1st-order BLCA clusters and the TCGA-defined clusters based on mutation and CNV data. *P*-value 0.128 in a *χ* ^2^ test of independence. This diagram compares the 125 samples that are defined in both cluster sets. (EPS 45294 kb)

Additional file 10: **Figure S9.** Screenshot of the interactive Tumor Map visualization, showing HOCUS applied to the TCGA Pancan-12 mutation data. Each point is one tumor sample, which we have color-coded by tissue type. A dotted box highlights the cluster of samples that have both PIK3CA and TP53 mutations, which are usually mutually exclusive. (EPS 751 kb)

Additional file 11: **Table S1.** *P*-values from *χ* ^2^ tests on all the data types. (XLSX 41 kb)

Additional file 12: **Figure S10:** (a) Pathology T stage of the HOCUS copy number clusters. (b) Enrichment of Gleason scores in the HOCUS clusters. Scores are normalized by column and color represents percentage of the cluster with a given combined Gleason score. (c) Boxplot of the number of lymph nodes each cluster’s samples have invaded. (EPS 221 kb)

Additional file 13: **Figure S11.** (a) Heatmaps of tumor image, where each heatmap shows one slice of the brain and is colored by the overlapping tumors between patients in the cohort before image filtering. Each heatmap shows one slice of the brain. From top left to bottom right, the slices start at the top of the head at show progressively lower portions of the brain. (b) Log change in tumor volume as MR images are filtered at different thresholds. (c) Lefthand plot shows the number of samples whose tumors are completely masked by filtering, and the righthand plot shows the per-patient tumor volume before and after filtering. Each dot represents one patient. (d) As in (a), these are heatmaps of the tumor images after filtering. (EPS 19672 kb)

Additional file 14: **Table S2.** Kernel similarity scores between each HOCUS feature space, survival in days, and age. (XLSX 610 kb)

Additional file 15: **Figure S12.** (a) Sagittal, coronal, and axial views of the tumors within each image cluster (b) Violin plots of tumors volumes for each cluster. (c) Comparison to molecular subtypes defined by TCGA. (d) Kaplan-Meier plot of image clusters, showing clusters 3 and 4 to have poorer overall survival. (e) Consensus clustering matrices for 2nd- and 3rd-order HOCUS clusters, connected by an alluvial diagram showing that the majority of patients in 2nd-order clusters 3 and 4 (the poor survivors) make up the 3rd-order cluster 3. (EPS 15887 kb)

Additional file 16: **Figure S13.** (a-c) Patients grouped on tumor volume and (d-f) by TCGA defined molecular subtypes for MR image patients. (a) Images of patient tumors grouped by tumor volume (b) molecular subtypes (c) KM survival. (d) Images of patient tumors grouped by molecular subtype, (e) tumor volume per group, (f) KM survival. (EPS 6584 kb)

Additional file 17: **Figure S14.** KM plot of survival when patients are grouped by anatomic location of the tumor. Annotations indicate laterality (right/left) and lobes (parietal, occipital, frontal, temporal). (JPG 51 kb)

Additional file 18: **Table S3.** *P*-values from *χ* ^2^ tests between image clusters of all types and clinical covariates. (XLSX 51 kb)

## Abbreviations

BLCA: Bladder Urothelial Carcinoma
BRCA: Breast invasive carcinoma
CNV: Copy number variation
GBM: Glioblastoma multiforme
HOCUS: Higher-Order Correlations to Uncover Subtypes
MNI: Montreal Neurological Institute 152
MR: Magnetic resonance
MRI: Magnetic resonance imaging
NMF: Non-negative matrix factorization
OV: High-grade serous ovarian cancer
PRAD: Prostate adenocarcinoma
TCGA: The Cancer Genome Atlas
