# Supplementary figures and images for: Revealing cancer subtypes with higher-order correlations applied to imaging and omics data
Source: BMC Med Genomics. 2017 Mar 31;10:20. doi: 10.1186/s12920-017-0256-3 (PMC5374737; doi:10.1186/s12920-017-0256-3)

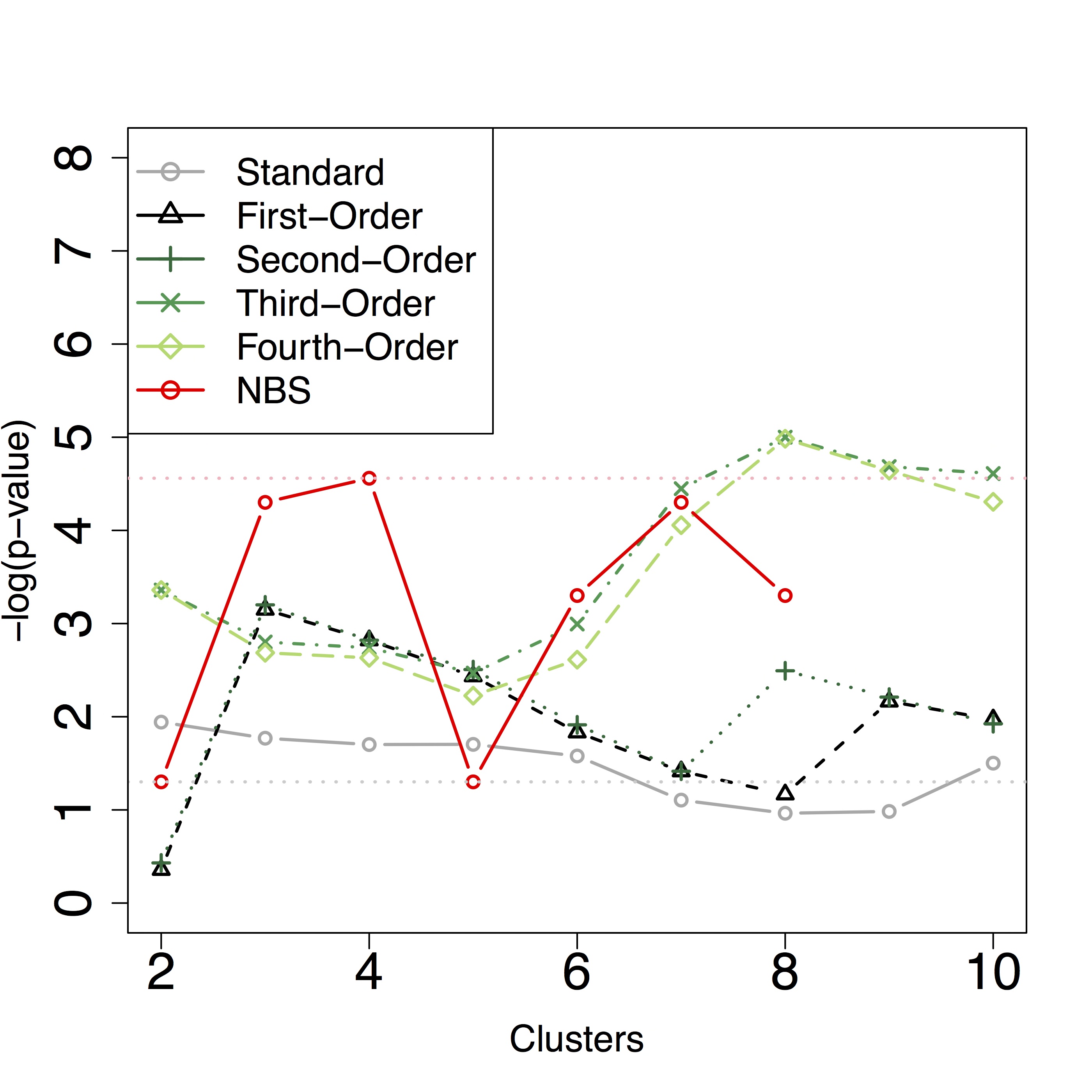

Supplement: Supplementary file 5 — Figure S5. Comparison to Network-Based Stratification [6] using the TCGA OV data used in their publication, and the same filtering. (JPG 528 kb) [file 12920_2017_256_MOESM5_ESM.jpg]

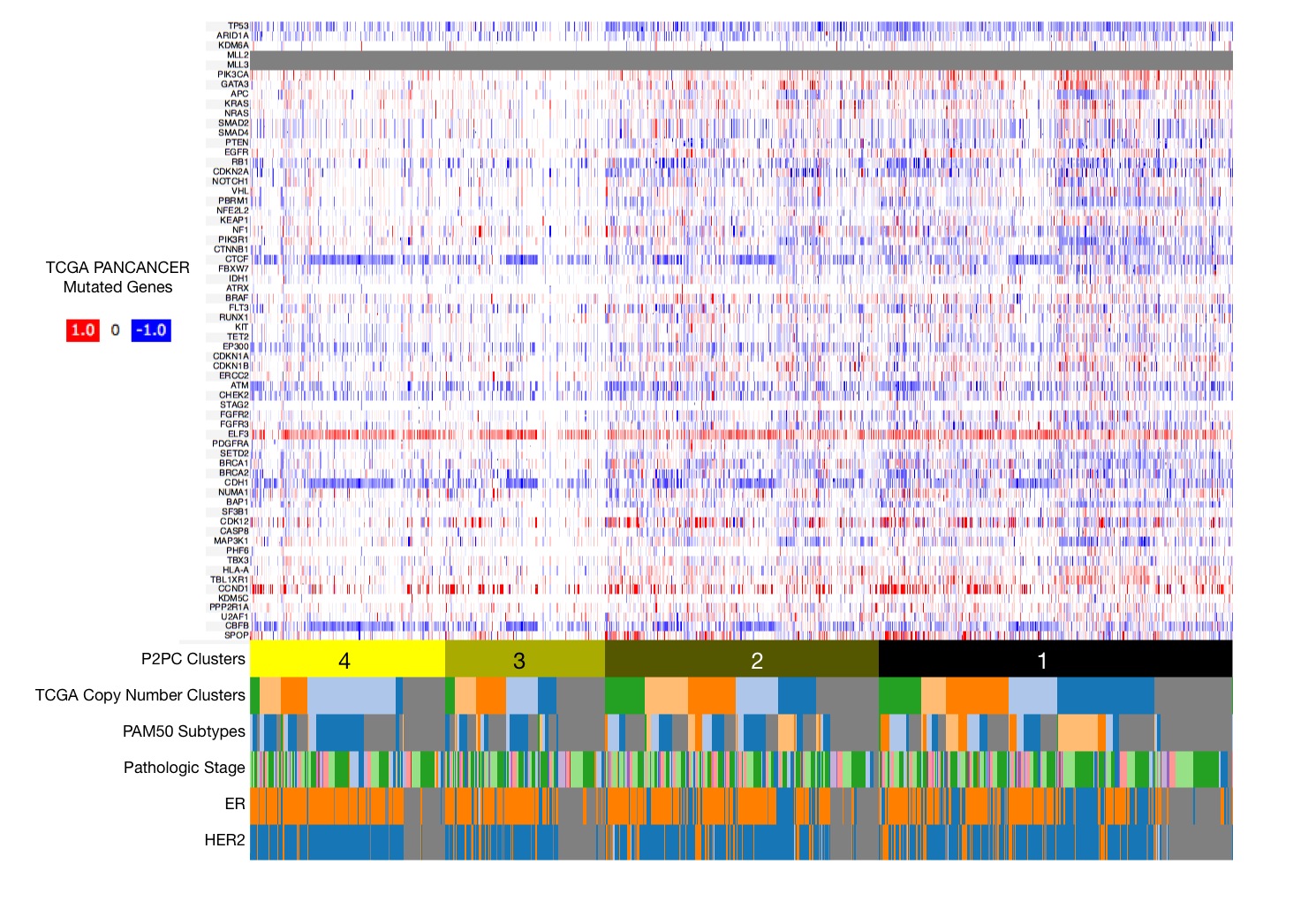

Supplement: Supplementary file 8 — Figure S7. Visualization of the BRCA copy number clusters and their correlation with the mutation-based subtypes from HOCUS. Heatmap made using the UCSC Cancer Genomics Browser [7], showing TCGA CNV subtypes and CNV alterations in the HOCUS clusters. (JPG 531 kb) [file 12920_2017_256_MOESM8_ESM.jpg]

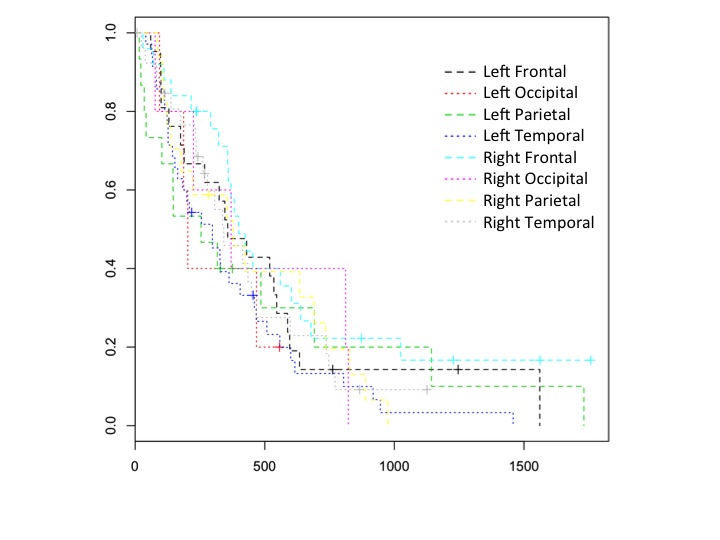

Supplement: Supplementary file 17 — Figure S14. KM plot of survival when patients are grouped by anatomic location of the tumor. Annotations indicate laterality (right/left) and lobes (parietal, occipital, frontal, temporal). (JPG 51 kb) [file 12920_2017_256_MOESM17_ESM.jpg]
