# Supplementary material for: Revealing cancer subtypes with higher-order correlations applied to imaging and omics data
Source: BMC Med Genomics. 2017 Mar 31;10:20. doi: 10.1186/s12920-017-0256-3 (PMC5374737; doi:10.1186/s12920-017-0256-3)
Supplement: Supplementary file 6 — Supplemental text. (DOC 132 kb) [file 12920_2017_256_MOESM6_ESM.doc]

**SUPPLEMENTAL TEXT**

for “Revealing Cancer Subtypes with Higher-Order Correlations Applied to Imaging and Omics Data”

## **SUPPLEMENTAL METHODS**

### Processing the Data

HOCUS can also be improved by filtering the data. In the main text we explore filtering MR image data. Mutations and copy number data may also benefit from filtering, for example excluding patients with more than 200 mutations as is often done in TCGA analysis. Even without filtering, HOCUS applies well to mutation data as well as MR image data.

MR images required high levels of filtering (Fig. S11) to mask out the non-brain regions of the images since brains have slightly different shapes and also because tumor appears in only white tissue, not grey. Similarly, many genes are mutated in only 1 sample in a cohort, which makes them difficult to use as a social connection; they are essentially friendless and can never contribute to building similarities between pairs of patients. Eliminating them would reduce similarity score inflation.

### 0.0.1 MR Image Preprocessing

The size of the MR images is prohibitive to analysis. We filtered the MR images to remove non-informative voxels prior to clustering. Because GBM occurs primarily in the white matter of the brain, most voxels elsewhere (*e.g.* gray matter) do not contain tumor. By filtering out voxel locations in which no patients have tumor occupancy, 80% of the voxels are eliminated without losing any information for subtyping.

After this step, 1 million voxels per sample remain, which is still a large number of features and impractical for use in most clustering algorithms. We therefore sought further filtering steps. We reason that voxels with little variation across the cohort – usually those in which only a few patients had tumors that overlapped the voxel – are uninformative for clustering patients into subtypes.

Thus, we retain only voxels with events in a minimum of
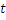
 patient samples. Setting
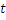
 too low yields an unmanageable number of uninformative voxels, while setting
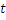
 too high masks out an appreciable amount of tumor for many of those samples. If
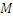
 is the samples-by-voxels matrix, then define
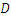
 to be the filtered matrix containing only voxels that appear in a minimum of
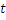
 patients; i.e. we set
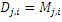
 if
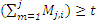
and otherwise it is set to *0*, where
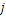
 is a sample and
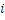
 is a voxel. The number of voxels retained for each sample
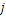
 is
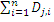
 and changes as a function of
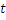
, which we plot in Fig. S11.

A minimum threshold of 15 tumor occurrences preserved voxels with an enrichment for high variability (high entropy) that would be informative for subtyping patients. Restricting to these voxels provided sufficient data for most of the patients in the cohort (Fig. S11) and did not seem to bias the set around any particular subset of tumors. Tumor voxels removed by this threshold are those that buttress the most dense tumor regions. By removing these voxels we minimize relationships defined by the uncommon tumor regions– in most patients these will be completely non-tumor, inflating the patient-patient similarity scores. This also forces the clustering methods to focus on the core of common tumor regions, which are of more interest in this study.

### 0.0.2 Alternative Similarity Metrics

We tested alternative similarity metrics on the MR Imaging data – TFIDF (Term Frequency Inverse Document Frequency) [[1]](https://paperpile.com/c/6JpY3k/dJnE), Jaccard similarity, and tumor volume scaled (Table S4, Fig. S1(f)). Many tumors were considered very similar when using Hamming similarity, due to the coincidence of pairs sharing mostly non-tumor voxels. We chose TFIDF weighting as an alternative metric because it can discount voxels that rarely overlap with tumor in the cohort. To avoid this, we add weighting to the voxel matching, using term (aka voxel) frequency [[1]](https://paperpile.com/c/6JpY3k/dJnE) such that in addition to summing the number of matched voxels, we scale the weight of the matches by the relative frequency of tumor in that voxel within the cohort. The similarity metric now also ignores tumor-free voxels when doing patient-patient comparisons, greatly reducing the number of voxels compared.

The two solutions are nearly identical except in two respects. First, TFIDF HOCUS disperses the Hamming HOCUS small-tumor cluster into the other 4 clusters. Second, it creates a new cluster with the 3 samples that have no visible tumor in the post-processed tumor data. TFIDF clusters are still correlated with tumor volume and lose the location-dependence of the Hamming HOCUS clusters (Fig. S1).

For the analysis described in the main text, the Hamming distance was used because the alternative metrics produced comparable solutions on the datasets considered and Hamming is simpler. However, in general, the choice of similarity metric can drastically alter analysis findings, and we recommend that users consider the structure of their data and pick an appropriate similarity metric. For example Jaccard similarity is often used in analyzing social networks. Using this metric, HOCUS finds 5 clusters that are volume dependent and molecular subtype independent clusters (Fig. S1). We investigated the association of each metric with the survival similarities of patient pairs. Note that the results on survival did not influence our choice of a metric, to avoid overfitting the analysis to clinical outcomes. Survival separation is comparable to the Hamming HOCUS clusters, however the most different (and best surviving) cluster is composed mostly of small tumors.

Table S4 shows the correlations between each similarity metric clustering solution and tumor volume, molecular subtype, and survival. Equations for calculating each similarity metric are also in this table. Fig. S1(f) shows patient cluster membership changes between all clustering solutions.

###

## **SUPPLEMENTAL RESULTS**

### 2nd-Order HOCUS GBM Imaging– Location-Finding

While appearing to be symmetric, clusters of patients on either side of the brain (Fig. S12(a)), clusters 4 and 5 are distinct from 3 in that there are 2 focal points of the tumors. Cluster 3 tumors show mid-region focus on the right side of the brain, whereas clusters 4 and 5 split into 2 clusters on the left side. Tumor focal points of the patient groups are on the edges of that region– cluster 5 patients have tumor higher in the brain whereas cluster 4 patients have tumor much lower and closer to the base of the skull. Thus we do not see symmetry in tumor growth based on brain hemisphere. Despite close physical proximity, clusters 4 and 5 have wildly different survival prognoses– cluster 5 having a long projected survival whereas clusters 3 and 4 have the worst in the cohort (Fig. S12(d)).

In previous works, Jain et al [[2]](https://paperpile.com/c/6JpY3k/FLFJ) and Liu et al [[3]](https://paperpile.com/c/6JpY3k/mj0u) find a correlation between volume of tumor in MR images and patient survival. Our data shows a similar trend. To illustrate this we divided the tumors groups based on tumor volume (Fig. S13(a)), finding poor prognosis for large tumors. HOCUS clusters show larger separation in survival than the volume-based groups (Table S3, log-rank test), indicating that tumor volume is one of many vital components of the image data. Furthermore, there is no distinction in survival separation between tumors grouped by the expert annotations of anatomic location (log-rank test, p-value 0.596, Fig. S14). HOCUS clusters span multiple locations specified by expert anatomic annotations.

There is little enrichment in HOCUS clusters of age, race, ethnicity, tumor status, gender, or surgical resection (Table S3). The clusters are also independent of tumor volume. Of note, even though the assay times at which Karnofsky Performance Scores (KPSs) were taken for TCGA samples are not documented, the volume-correlated first-order HOCUS clusters exhibit a significant association with KPS.

### 0.0.3 HOCUS of TCGA Pancan-12 Mutations

We applied HOCS to the TCGA Pancan-12 mutation data to show that HOCUS performs well on larger datasets as well. This was then integrated into an interactive visualization tool, Tumor Map. From this map we identify an estrogen–signaling cluster which contains the BRCA luminal samples, and another cluster composed of samples with mutations in both TP53 and PIK3CA (Fig S9).

### 0.0.4 HOCUS of TCGA BRCA Mutations

Because of their demonstrated clinical import, BRCA subtypes are often defined using gene expression data. It has been shown that clusters based on mRNA transcription data readily identify luminal-like from the more aggressive basal-like tumors. While luminal tumors tend to be associated with the expression and presentation of the estrogen receptor, the basals tend to be less differentiated and lack this and other hormone receptors (such as progesterone receptor).

The BRCA subtypes identified by the second-order HOCUS algorithm are distinct from the more established expression-based subtypes (*e.g.* basals and luminals). However, since the mutation-based clusters provide independent information for predicting patient survival in a multivariate analysis (
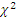
 test, P < 5.428232e−17), it is critical to incorporate the SNV-based subtypes into categorization as has been shown by [[4]](https://paperpile.com/c/6JpY3k/2l5z). As might be expected, the first major split of the data separates tumors with elevated mutation rates (cluster 1 in Fig. S6) from those with fewer mutations, such as cluster 3 that has the least. Cluster 4 is predominantly luminal A while the other clusters contain a mixture of the expression-based subtypes (see Fig. S6). The samples in the cluster with elevated mutation rate are enriched for mutations in TP53 (50%), which may be an early event in these tumors enabling global loss of genome integrity. Consistent with this statement is the observation that these tumors also have the highest levels of copy number alterations as well (Fig. S7). The hyper-mutated group also contains PIK3CA mutations that are often mutually-exclusive with TP53. In addition, the cluster contains mutations in what are thought to be passenger genes frequently mutated in some cancers possibly due to their location with respect to late replication fork timing during mitosis [[5]](https://paperpile.com/c/6JpY3k/qdit) such as TTN and MUC16. Taken together, the results suggest cluster 1 represents more advanced tumors.

On the other hand, cluster 3 not only contains the fewest mutations but also lacks mutations in TP53 or PIK3CA. Nearly all normal-like tumors fall into this group, which recapitulates the expression-based designation for these tumors. Samples in cluster 3 have mutations characteristic of luminal-A tumors such as in the PI3-kinase pathway (MAP3K1 and CDH1) and GATA3. Interestingly, several tumors classified as basal using expression data fall into this group. It would be interesting to determine if these tumors do indeed have TP53 mutations that were not detected through the TCGA’s whole exome sequencing analysis (e.g. through regulatory mutations in promoter elements) or through other mechanisms (*e.g.* epigenetic silencing) or genes that can influence TP53 function.

The HOCUS method identifies a PIK3CA mutated group (cluster 4), a clear hallmark of luminal-A breast cancers. This group also has enrichment for other PI-3-kinase pathway mutations such as in MAP3K1 and CDH1, underscoring the selective pressure to enhance signaling in this growth-related pathway for this tumor type.

Cluster 2 samples are notable for mutational frequencies equal to that across the cohort, and yet having neither TTN nor PIK3CA mutations. Approximately 30% of these samples have TP53 mutations.

**SUPPLEMENTAL REFERENCES**

[1. Sparck Jones K. A STATISTICAL INTERPRETATION OF TERM SPECIFICITY AND ITS APPLICATION IN RETRIEVAL. Journal of Documentation. 1972;28:11–21.](http://paperpile.com/b/6JpY3k/dJnE)

[2. Jain R, Poisson L, Narang J, Gutman D, Scarpace L, Hwang SN, et al. Genomic mapping and survival prediction in glioblastoma: molecular subclassification strengthened by hemodynamic imaging biomarkers. Radiology. 2013;267:212–20.](http://paperpile.com/b/6JpY3k/FLFJ)

[3. Liu TT, Achrol AS, Mitchell LA, Du WA, Loya JJ, Rodriguez SA, et al. Computational Identification of Tumor Anatomic Location Associated with Survival in 2 Large Cohorts of Human Primary Glioblastomas. AJNR Am J Neuroradiol. 2016;37:621–8.](http://paperpile.com/b/6JpY3k/mj0u)

[4. Ciriello G, Miller ML, Aksoy BA, Senbabaoglu Y, Schultz N, Sander C. Emerging landscape of oncogenic signatures across human cancers. Nat Genet. 2013;45:1127–33.](http://paperpile.com/b/6JpY3k/2l5z)

[5. Garraway LA, Lander ES. Lessons from the cancer genome. Cell. 2013;153:17–37.](http://paperpile.com/b/6JpY3k/qdit)

[6. Hofree M, Shen JP, Carter H, Gross A, Ideker T. Network-based stratification of tumor mutations. Nat Methods. 2013;10:1108–15.](http://paperpile.com/b/6JpY3k/7u2S)

[7. Goldman M, Craft B, Swatloski T, Cline M, Morozova O, Diekhans M, et al. The UCSC Cancer Genomics Browser: update 2015. Nucleic Acids Res. 2015;43 Database issue:D812–7.](http://paperpile.com/b/6JpY3k/YQaI)
